# Comparing Self-Reported Sugar Intake With the Sucrose and Fructose Biomarker From Overnight Urine Samples in Relation to Cardiometabolic Risk Factors

**DOI:** 10.3389/fnut.2020.00062

**Published:** 2020-05-06

**Authors:** Stina Ramne, Nicola Gray, Sophie Hellstrand, Louise Brunkwall, Sofia Enhörning, Peter M. Nilsson, Gunnar Engström, Marju Orho-Melander, Ulrika Ericson, Gunter G. C. Kuhnle, Emily Sonestedt

**Affiliations:** ^1^Department of Clinical Sciences Malmö, Faculty of Medicine, Lund University, Malmö, Sweden; ^2^Department of Food and Nutritional Sciences, School of Chemistry, Food and Pharmacy, University of Reading, Reading, United Kingdom; ^3^Department of Internal Medicine, Skåne University Hospital, Malmö, Sweden

**Keywords:** added sugar intake, nutritional biomarkers, urinary sucrose and fructose, overnight urinary sugars, cardiometabolic risk factors

## Abstract

Studies on sugar intake and its link to cardiometabolic risk show inconsistent results, partly due to dietary misreporting. Cost-effective and easily measured nutritional biomarkers that can complement dietary data are warranted. Measurement of 24-h urinary sugars is a biomarker of sugar intake, but there are knowledge gaps regarding the use of overnight urine samples. We aim to compare (1) overnight urinary sucrose and fructose measured with liquid chromatography-tandem mass spectrometry, (2) self-reported sugar intake measured with web-based 4-day food records, (3) their composite measure, and (4) these different measures' (1–3) cross-sectional associations with cardiometabolic risk factors in 991 adults in the Malmö Offspring Study (18–69 years, 54% women). The correlations between the reported intakes of total sugar, added sugar and sucrose was higher for urinary sucrose than fructose, and the correlations for the sum or urinary sucrose and fructose (U-sugars) varied between *r*≈0.2–0.3 (*P* < 0.01) in men and women. Differences in the direction of associations were observed for some cardiometabolic risk factors between U-sugars and reported added sugar intake, as well as between the sexes. In women, U-sugars, but not reported added sugar intake, were positively associated with systolic and diastolic blood pressure and fasting glucose. Both U-sugars and added sugar were positively associated with BMI and waist circumference in women, whereas among men, U-sugars were negatively associated with BMI and waist circumference, and no association was observed for added sugar. The composite measure of added sugars and U-sugars was positively associated with BMI, waist circumference and systolic blood pressure and negatively associated with HDL cholesterol in women (*P* < 0.05). Conclusively, we demonstrate statistically significant, but not very high, correlations between reported sugar intakes and U-sugars. Results indicate that overnight urinary sugars may be used as a complement to self-reported dietary data when investigating associations between sugar exposure and cardiometabolic risk. However, future studies are highly needed to validate the overnight urinary sugars as a biomarker because its use, instead of 24-h urine, facilitates data collection.

## Introduction

Sugars have received increasing attention in recent decades and have been linked to metabolic syndrome and related conditions and diseases (obesity, type 2 diabetes and cardiovascular disease) (1–3). However, the strength of the available evidence is weak (4), and the inconsistent results might partly be explained by difficulties in measuring sugar intake as an exposure. Misreporting through self-reported dietary assessment methods is a challenge that complicates the conclusions of epidemiological investigations of health risks associated with high sugar consumption. Hence, there is a need to identify objective measurements of dietary intake in the form of nutritional biomarkers to complement subjective self-reported data (5). It should also be emphasized that with this need for nutritional biomarkers follows an almost equally important need for these biomarkers to be relatively cost-effective and easily measured.

The measurement of 24-h urinary sucrose and fructose as a predictive biomarker for sugar intake was first recognized after its dose-response relationship was demonstrated through controlled sugar intake and its validity to estimate sugar intake (after *ad libitum* intake) was confirmed (6). Thereon, this biomarker has been compared against several different dietary assessment methods (7, 8), e.g., correlation of *r* = 0.21 with a 4-day food record (8). As compared to the predictive 24-h urinary sugar biomarker, the concentration biomarker from spot or overnight urinary sugar samples (9, 10) is substantially easier to collect but has only been compared with reported sugar intake in three previous studies (two in the same cohort) (11–13). Only one of these studies, which was performed in children, reported correlation coefficients between the spot morning urinary sugar levels and reported sugar intake (*r* = 0.25) (13). In the other cohort, higher urinary sucrose levels (from any time spot urine samples) were associated with an increased risk of being overweight, whereas higher self-reported sugar intake was associated with a decreased risk (12).

The principle behind this biomarker is based on the understanding that very small amounts of sucrose can evade hydrolysis by sucrase and be absorbed in the jejunum as a disaccharide instead of being cleaved into glucose and fructose (10, 14). Fructose, either directly from the diet or as hydrolyzed sucrose, is transported to the liver and only small amounts can evade the hepatic metabolism and remain in the circulation. In the circulation, sucrose and fructose, unlike glucose, are not hormonally regulated by insulin, and hence, non-metabolized sucrose and fructose are excreted in the urine (15). At most, ~0.05% of consumed sucrose and fructose is excreted in the urine and detected in 24-h samples, but this small amount correlates very well with sugar intake under controlled dietary intake and urination conditions (*r* = 0.88) (6). This correlation exists even though the dietary and urinary sugars reflect somewhat different factors: consumed and absorbed sugars. However, there is a lack of knowledge on the performance of this biomarker in free living populations and in overnight urine instead of 24-h urine samples, which means that both the biomarker and self-reported dietary data in this study are subject to individual uncertainties.

This biomarker from non-24-h urine samples classifies as a so-called concentration biomarker, and therefore lacks the ability to predict true sugar intake and to use for regression calibration (10, 16). However, Freedman et al. (17, 18) have proposed that combining self-reported intake with concentration biomarkers into composite measures is a way to improve investigation of diet-disease relationships.

The objective of this study was to compare the measurement of sucrose and fructose in overnight urine samples, self-reported sugar intake and their combination, as well as to assess and compare their associations with cardiometabolic risk factors.

## Materials and Methods

### Study Design and Subjects

**Figure 1:**
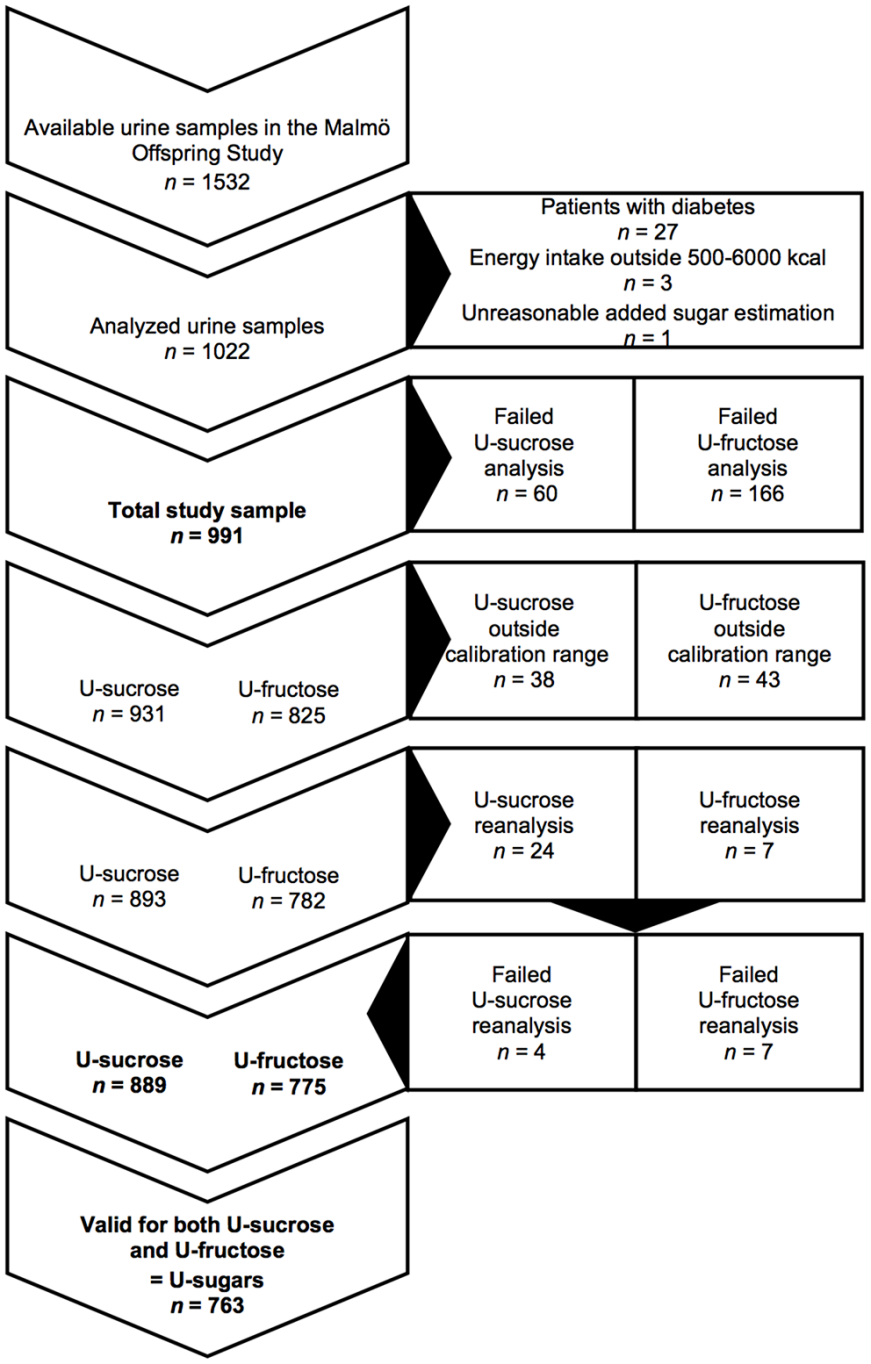
Flow chart of participants and urine samples in the Malmö Offspring Study. U-sucrose, urinary sucrose; U-fructose, urinary fructose; U-sugars, sum of urinary sucrose and fructose.

The Malmö Offspring Study (MOS) is a prospective cohort comprised of adult children and grandchildren of participants from the Malmö Diet and Cancer-Cardiovascular Cohort (MDC-CC), which was conducted in the 1990s. The individuals comprising MOS were recruited through invitation letters beginning in 2013. The eligibility criteria were a minimum of 18 years of age and at least one parent or grandparent who participated in the MDC-CC. The participants visited the research clinic twice (~1 week apart) for clinical examinations, collection of biological samples and instructions on how to fill in a lifestyle questionnaire and maintain a 4-day web-based food record at home (19). Participants started recording their diet prospectively the day after their first visit and brought their overnight urine samples on the morning of the second visit. For the present cross-sectional study, from the first 1,532 urine samples collected in the MOS, we selected those from all the non-diabetic participants with complete dietary data and a reported energy intake within the range of 500–6,000 kcal/d (Figure 1). The MOS received ethical approval from the Regional Ethics Committee in Lund (Dnr.2012/594) and all the participants signed a written informed consent form prior to participation, all in accordance with the Declaration of Helsinki.

### Dietary Data

Dietary data were collected using the Riksmaten2010 method, developed by the Swedish National Food Agency, which involves an online 4-day web-based food record (20). Each participant started recording their diet on the day after the first visit to the research clinic to ensure representation of all days of the week among the studied population. The participants were instructed to record everything they consumed for four consecutive days. The portion size was estimated using photographs of different portion sizes, and the food record was linked to the food database of the Swedish National Food Agency.

Data on total mono- and disaccharides and sucrose were obtained from the food database. Total sugars (g/d) were calculated by summing all mono- and disaccharides (which includes glucose, fructose, galactose, sucrose, lactose and maltose), and the total sugar density (g/1,000 kcal) was calculated by dividing the total sugar intake by the energy intake/1,000. The level of added sugars, as defined by the European Food Safety Authority and the Nordic Nutrition Recommendations: “The term “added sugars” refers to sucrose, fructose, glucose, starch hydrolysates (glucose syrup, high-fructose syrup) and other isolated sugar preparations used as such or added during food preparation and manufacturing.” (21, 22) (including isolated sugar preparations such as honey and syrup), was estimated by subtracting naturally occurring monosaccharides and sucrose in fruits and berries (10 g/100 g), vegetables (3 g/100 g), and juices (8 g/100 g) from the sum of the reported intake of monosaccharides and sucrose (assuming that lactose and maltose are not added to foods). The following formula was used for the estimation (all intake variables are expressed in g/day): added sugar = monosaccharides + sucrose—(fruit and berries × 0.1 + vegetables × 0.03 + juice × 0.08). The resulting value was transformed into the percentage of non-alcoholic energy intake (E%). The total sugar density is expressed in g/1,000 kcal, whereas the added sugar density is expressed in E% to facilitate comparisons with previous studies. The investigated sugar sources were desserts (desserts, cakes, cookies, pastries and ice cream), sweets (sweets, chocolate and bars), toppings expressed in servings/day (1 serving of table sugar, syrup or honey = 10 g, 1 serving of jam, marmalade or jelly = 20 g), sugar-sweetened beverages (SSBs; soft drinks, non-carbonated sugar-sweetened drinks, chocolate drinks), fruits (fruits and berries including dried and preserved) and juices (fruit and vegetable juices). One subject outlier was excluded from the statistical analysis due to an extremely high reported juice intake, which resulted in an unreasonable estimation of added sugar intake; and thus, the total study sample comprised 991 subjects (Figure 1). The added sugar intake (E%) was the dietary variable primarily investigated in this study because that is what is of most interest in terms of cardiometabolic risk. This is despite the fact that the measured urinary biomarker cannot distinguish between naturally occurring and added sucrose and fructose, but the alternative, i.e., investigation of the total sugar intake, does not perfectly reflect the biomarker either because both lactose and maltose form a substantial part of the total sugars.

### Urinary Data

#### Collection of Overnight Urine Samples

Comprehensive instructions to ensure a standardized urine collection procedure were provided on the first visit to the research clinic. Overnight urine was collected on the morning of the second research visit. The instructions were to empty the bladder before bedtime and collect all urine thereafter during the night and all of the first morning urine in a plastic bottle while fasting. At the clinic, the urine samples were stored in a refrigerator for a maximum of 4 h before being transferred to the laboratory, where they were aliquoted and relocated to a −80°C freezer.

#### Preparation of Calibration Standards, Internal Standards and Quality Control

Calibration standards of sucrose and fructose (Sigma Aldrich, Gillingham, UK) were prepared ranging from 0.1 to 500 μmol/L. Stable isotope-labeled internal standard solution was prepared in acetonitrile containing ^13^C_12_-sucrose at 4 μg/mL and ^13^C_6_-fructose at 10 μg/mL. Quality controls (QCs) of 1 μmol/L (low QC), 7.5 μmol/L (medium QC), and 75 μmol/L (high QC) were analyzed in duplicate throughout each batch of samples. The precision and accuracy of the analysis were assessed by determining the replicates of the low, medium and high QC samples across all batches of samples.

#### Liquid Chromatography-Tandem Mass Spectrometry (LC-MS/MS)

The urine samples were stored at−80°C until, thawed at 4°C and diluted with the internal standard mix. LC-MS/MS analysis was performed using an Acquity UPLC system (Waters, Milford, MA, USA), coupled to a Quattro Ultima tandem quadrupole mass spectrometer (Micromass, Manchester, UK). The mass spectrometer was operated through electrospray ionization in positive ion mode using multiple reaction monitoring mode. In total, 226 samples were not successfully analyzed, and 81 samples were outside the calibration range and were thus excluded from the analysis. Out of those 81 samples, those above the calibration range (24 sucrose samples and 7 fructose samples) were reanalyzed at a 4-fold-higher dilution and a calibration range extending to 1,000 μmol/L. These re-analyses were successfully performed and resulted in re-inclusion for the sucrose samples (with the exception of 4 samples), but not with fructose (Figure 1).

#### Adjustment for Urine Dilution

To adjust for urine dilution, the urinary sugars concentrations were expressed as ratios to the urine osmolality (U-osm, mOsm/kg H_2_O), i.e., in units of (μmol·L^−1^)/(mOsm·kg^−1^). U-osm was selected for dilution adjustment over urinary creatinine because the latter could be associated with body mass index (BMI) and could consequently induce a false association between urinary sugars and BMI. U-osm was measured using an i-Osmometer basic (Löser, Germany). The osmolality-adjusted urinary sucrose (U-sucrose) and fructose (U-fructose) were also added together and investigated as their sum (U-sugars). U-sugars was correlated (*r* = 0.95, *P* < 0.001) with the sum of urinary sucrose and fructose adjusted for creatinine. The osmolality-adjusted urinary sugar variables are used throughout this paper unless stated otherwise.

### Data on Cardiometabolic Risk Factors

During the visits to the research clinic, anthropometrics and blood pressure were measured, and fasting blood samples were collected by a research nurse. Weight was measured using either a calibrated balance beam or a digital scale with the participant wearing light clothing. Height was measured to the nearest centimeter using a stadiometer. BMI was calculated as weight (kg)/height (m)^2^ (19). Waist circumference was measured between the lowest rib margin and the iliac crest with the participant in a standing position. Resting blood pressure (BP) was assessed while lying after 10 min rest as the mean from two measurements. Fasting blood samples were drawn and plasma was analyzed directly for fasting glucose using HemoCue Glucose 201^+^ (HemoCue AB, Sweden) and within 4 h for total cholesterol, triglycerides and high-density lipoprotein (HDL) cholesterol using the Cobas system (Roche Diagnostics, Germany). Low-density lipoprotein (LDL) cholesterol was calculated using the Friedewald equation.

### Data on Confounding Factors

Data on potential confounders were collected via a lifestyle questionnaire. Leisure-time physical activity (LTPA) was assessed using a four-level scale ranging from sedentary to regular activity (≥3 × 30 min/week). Smoking status was categorized as never smoked, ex-smoker, irregular smoker, and regular smoker. Alcohol consumption habits were assessed on a five-level scale from never to ≥4 times/week, and their education level was categorized as compulsory school, upper secondary school and university degree. Data on medication use were also self-reported via the lifestyle questionnaire. The relative estimated glomerular filtration rate (e-GFR) was estimated using the revised Lund-Malmö equation (23).

Misreporting of energy intake was evaluated according to Goldberg and Black's cutoffs for misreporting of energy intake based on a two-standard-deviations discrepancy between individual physical activity levels and the ratio of energy intake to the basal metabolic rate (24). Individual physical activity levels were obtained from the Riksmaten2010 based on physical activity at work and LTPA (both assessed using a four-level scale ranging from sedentary to heavy manual labor/exercise ≥3 × 30 min/week). To enable comparisons with the ratio of energy intake to basal metabolic rate, basal metabolic rate was calculated using the Oxford equations by taking sex, age, and weight into account (22, 25).

### Statistical Analysis

All statistical analyses were performed using Stata/SE (version 15; StataCorp LLC, USA). The urinary sugar variables were skewed even after adjustment for U-osm and were therefore log_10_-transformed. Since sex differences were observed, the statistical analyses were mainly performed divided by sex.

The baseline characteristics were evaluated separately for men and women over quintiles of U-sugars and 6 categories of added sugar intake, namely, ≤ 5E, >5- ≤ 7.5E, >7.5- ≤ 10E, >10- ≤ 15E, >15- ≤ 20E, and >20E%, which were previously investigated in relation to mortality in the Malmö Diet and Cancer Study (26). Categorical variables were expressed as percentages and continuous variables were expressed and mean (SD) or median (IQR), dependent on their distribution.

Partial correlation analysis adjusting for energy intake, age, sex, and BMI was performed between the different urinary sugar variables and the reported dietary sugar variables to evaluate the agreement between the two measurement methods. An alluvial plot was created to visualize the agreement between the 6 added sugar intake categories and the quintiles of U-sugars. To assess misclassification, equal groups are necessary and for this purpose sex-specific quartiles were used for crosstabulation of the U-sugars and reported added sugar intake.

We assume no mediation through the urinary sugar biomarker in the potential association between sugar intake and cardiometabolic disease. Therefore, according to reasoning by Freedman et al. (17) the combination of U-sugars and reported added sugar intake (E%) into one composite measure of exposure was obtained using both the principal component (PC) method and the Howe's method. In the PC method, in the case of only two variables that are positively correlated (such as in this situation), the first PC is proportional to the sum of the two variables, each divided by their own standard deviations. In Howe's method, all U-sugar values and added sugar values were ranked, and the ranks were summed (17).

Linear regression was used to examine the associations of U-sugars, reported added sugar intake (E%) and their composite measures with cardiometabolic risk factors. In the regression models, model 1 was adjusted for age and sex [and total energy intake for the analyses of added sugar intake (E%) and the composite measures, i.e., the multivariate nutrient density model was used for energy adjustment (27)], and model 2 was additionally adjusted for educational level, LTPA, smoking status, alcohol consumption habits and fiber density (g/1,000 kcal). The regressions with total cholesterol, triglycerides, HDL and LDL cholesterol were also adjusted for the usage of lipid-lowering drugs, and the regressions with systolic and diastolic BP were also adjusted for the usage of antihypertensive drugs. The interaction with sex was evaluated in all regression analyses. In sex-specific analyses, interactions with obesity (BMI≥30 or BMI <30) were evaluated for U-sugars, and interactions with energy underreporting (yes or no) and obesity were evaluated for added sugar intake.

To further understand the relationships, an attempt to identify and map out all potential and measured predictors of U-sugars was performed through partial correlation analyses for men and women separately. The multivariate partial correlation model was determined through stepwise backward linear regression. All covariates were added simultaneously to the linear regression model, and the covariate with the highest *P*-value was excluded from the model in a stepwise manor until all covariates were deemed statistically significant. The investigated variables included added sugar intake; intake of desserts, sweets, toppings, SSBs, fruit, and berries, and juice; educational level; smoking status; alcohol consumption habits; LTPA; BMI; waist circumference; systolic BP; fasting glucose; U-osm; and e-GFR. We investigated U-sugars unadjusted for U-osm. Instead, U-osm was included as a covariate. All of these sex-specific partial correlations and multivariate linear regressions were adjusted for energy intake and age. A significance level of α = 0.05 was applied and corrections for multiple testing were not performed. Therefore, the presented *P*-values should be interpreted with caution.

## Results

### Baseline Characteristics

Among the total study sample of 991 participants with complete dietary data, we obtained valid measurements within the calibration range for U-sucrose [median 32.7 μmol/L (12.6–85.7)] from 889 participants and for U-fructose [median 18.0 μmol/L (7.4–44.0)] from 775 participants (not adjusted for U-osm). In total, 763 participants presented valid measurements for both U-sucrose and U-fructose (Figure 1). The mean age of the cohort was 39 years (range 18–69). As observed in Tables 1, 2, the lowest mean age was seen in the highest groups of both added sugar and U-sugars. A higher percentage of women were seen among the low groups of both added sugar and U-sugars. Among those reporting high added sugar intake, we observed lower proportions of energy underreporters, high consumers of alcohol and individuals with regular LTPA ≥3 × 30 min/week in both men and women. In those with high U-sugars, the proportions with a university degree appeared to be lower. High reported energy intake was observed among those reporting high added sugar intake, but not in those with high U-sugars. Higher U-osm was observed in women with higher U-sugars. Intake of most sugar-rich foods and beverages appears to increase with increasing U-sugars, while intake of fruit appears to decrease. Although, a substantial part of zero-consumption has been reported for some of the sugar-rich foods and beverages (Table 2).

**Table 1:** Baseline characteristics of women and men in the Malmö Offspring Study across 6 categories of added sugar intake, E%.

**Added sugar intake, E%**	**≤5E%**	**>5E% to ≤7.5E%**	**>7.5E% to ≤10E%**	**>10E% to ≤15E%**	**>15E% to ≤20E%**	**>20E%**
**Women (*****n*** **=** **533)**						
*n* (% women)	13 (34.2)	50 (53.2)	100 (56.5)	192 (52.3)	119 (58.1)	59 (53.6)
University degree, %	60.0	45.8	50.0	48.1	41.7	49.1
LTPA ≥3 × 30 min/wk, %	46.2	38.0	21.0	35.9	35.3	20.3
Current smokers, %	0.0	14.6	11.0	8.15	13.8	19.6
Alcohol consumed >twice/wk, %	40.0	41.7	47.8	26.1	22.9	8.93
Energy underreporters, %	69.2	36.0	31.0	30.2	20.2	28.8
Age, y	39.3 (15)	45.7 (13)	43.2 (12)	39.7 (13)	37.6 (14)	32.5 (11)
BMI, kg/m^2^	22.2 (1.8)	24.7 (3.2)	25.2 (5.0)	25.0 (4.9)	24.1 (4.3)	26.1 (6.8)
Energy intake, kcal/d	1348 (402)	1569 (398)	1782 (450)	1833 (486)	1894 (493)	1920 (564)
U-osm, mOsm/kg	556 (325)	535 (218)	563 (233)	578 (231)	597 (229)	675 (255)
**Men (*****n*** **=** **458)**						
*n* (% men)	25 (65.8)	44 (46.8)	77 (43.5)	175 (47.7)	86 (42.0)	51 (43.4)
University degree, %	26.1	23.1	41.8	41.8	21.1	31.3
LTPA ≥3 × 30 min/wk, %	36.0	34.1	36.4	29.7	22.1	19.6
Current smokers, %	8.33	15.4	7.46	9.87	17.8	12.5
Alcohol consumed >twice/wk, %	25.0	53.9	46.3	39.7	28.8	21.7
Energy underreporters, %	64.0	45.5	46.8	30.3	24.4	25.5
Age, y	39.4 (11)	42.2 (15)	41.1 (13)	38.5 (13)	38.2 (13)	37.7 (13)
BMI, kg/m^2^	27.2 (4.1)	25.9 (3.3)	26.2 (2.9)	25.9 (4.2)	25.9 (4.3)	26.3 (4.1)
Energy intake, kcal/d	1991 (779)	2014 (532)	2127 (532)	2389 (672)	2440 (664)	2497 (669)
U-osm, mOsm/kg	695 (260)	660 (236)	683 (249)	737 (249)	689 (264)	764 (286)

*The categorical variables are expressed as percentages. The continuous variables are expressed as the means (SDs)*.

*LTPA, leisure-time physical activity; BMI, body mass index; U-osm, urine osmolality*.

**Table 2:** Baseline characteristics of women and men in the Malmö Offspring Study across quintiles (Q1–Q5) of U-sugars, (μmol·L^−1^)/(mOsm·kg^−1^).

	**Q1**	**Q2**	**Q3**	**Q4**	**Q5**
**U-sugars, mean**	**0.02**	**0.05**	**0.09**	**0.17**	**0.43**
**(range), (μmol·L^**−1**^)/(mOsm·kg^**−1**^)**	**(0.01–0.04)**	**(0.04–0.07)**	**(0.07–0.12)**	**(0.12–0.26)**	**(0.26–3.85)**
**Women (*****n*** **=** **412)**					
*n* (% women)	75 (49.0)	76 (50.0)	70 (45.8)	96 (62.8)	95 (62.5)
University degree, %	50.7	60.0	44.8	50.0	36.0
LTPA≥3 × 30 min/wk, %	29.3	35.5	31.4	25.0	26.3
Current smokers, %	7.04	8.45	10.5	17.4	13.5
Alcohol consumed >twice/wk, %	29.6	32.4	32.8	39.5	21.4
Energy underreporters, %	32.0	25.0	22.9	25.0	27.4
Age, y	41.1 (14)	42.2 (12)	40.1 (13)	39.8 (13)	36.9 (13)
BMI, kg/m^2^	24.4 (4.17)	25.2 (4.76)	24.6 (4.18)	24.6 (4.27)	25.5 (5.63)
U-osm, mOsm/kg	540 (236)	568 (229)	636 (250)	592 (239)	653 (244)
Energy intake, kcal/d	1,782 (508)	1,869 (481)	1,840 (443)	1,835 (500)	1,864 (536)
Sucrose, g/d	33.0 (20.3)	38.2 (23.7)	35.7 (19.2)	41.2 (23.9)	45.9 (27.8)
Total sugar, g/d	73.7 (30.4)	83.1 (31.2)	76.2 (30.5)	84.3 (35.2)	90.5 (38.4)
Total sugar density, g/1,000 kcal	41.3 (11.3)	44.6 (12.4)	41.1 (12.8)	45.5 (14.5)	48.3 (14.6)
Added sugar, E%	11.6 (4.98)	12.8 (4.50)	12.1 (4.92)	13.6 (5.85)	15.0 (5.42)
Desserts, g/d^a^	25.3 (10.0, 53.1)	28.1 (10.6, 69.8)	35.4 (21.3, 59.5)	38.1 (11.9, 73.1)	37.5 (12.8, 75.0)
Sweets, g/d^a^	10.0 (0.5, 20.8)	13.4 (3.5, 28.9)	13.8 (3.5, 37.0)	15.5 (4.3, 35.9)	13.8 (0.0, 50.0)
Toppings, servings/d^a^	0.24 (0.0, 0.54)	0.05 (0.0, 0.84)	0.0 (0.0, 0.59)	0.26 (0.0, 0.71)	0.0 (0.0, 0.54)
SSBs, g/d^a^	0.0 (0.0, 50.0)	0.0 (0.0, 75.0)	0.0 (0.0, 125)	25.0 (0.0, 129)	0.0 (0.0, 150)
Juice, g/d^a^	0.0 (0.0, 75.0)	0.0 (0.0, 90.6)	0.0 (0.0 (75.0)	0.0 (0.0, 87.5)	0.0, 0.0, 75.0)
Fruits, g/d^a^	103 (39.0, 170)	95.1 (55.8, 169)	81.1 (47.2, 128)	82.3 (32.6, 152)	75.6 (24.5, 155)
**Men (*****n*** **=** **351)**					
*n* (% men)	78 (51.0)	76 (50.0)	83 (54.3)	57 (37.3)	57 (37.5)
University degree, %	33.3	50.8	30.0	25.5	22.7
LTPA ≥3 × 30 min/wk, %	32.1	26.3	28.9	28.1	29.8
Current smokers, %	7.04	8.82	18.1	6.38	15.9
Alcohol consumed >twice/wk, %	40.3	48.5	40.9	36.2	27.3
Energy underreporters, %	39.7	36.8	33.7	31.6	21.1
Age, y	39.7 (11.0)	41.5 (12.8)	39.9 (14.5)	39.1 (13.7)	36.9 (14.3)
BMI, kg/m^2^	27.5 (4.52)	25.9 (3.53)	25.8 (3.61)	26.4 (3.98)	25.5 (4.23)
U-osm, mOsm/kg	740 (264)	698 (262)	692 (245)	689 (260)	781 (230)
Energy intake, kcal/d	2,278 (707)	2,297 (670)	2,370 (624)	2,285 (575)	2,479 (698)
Sucrose, g/d	36.6 (29.3)	47.4 (31.7)	47.0 (25.9)	50.3 (33.7)	66.8 (39.9)
Total sugar, g/d	79.9 (40.6)	93.7 (46.3)	96.7 (41.0)	97.7 (47.4)	113 (49.3)
Total sugar density, g/1,000 kcal	34.2 (12.0)	40.5 (13.6)	40.5 (12.9)	42.0 (16.3)	45.3 (14.5)
Added sugar, E%	10.6 (4.94)	12.5 (5.06)	13.0 (5.06)	13.8 (5.51)	15.4 (6.15)
Desserts, g/d^a^	27.5 (0.0, 57.5)	30.0 (6.3, 63.5)	38.0 (10.0, 72.5)	33.8 (10.0, 72.5)	56.3 (15.0, 94,8)
Sweets, g/d^a^	0.0 (0.0, 20.5)	7.8 (0.0, 20.8)	11.3 (0.0, 26.3)	9.0 (0.0, 35.0)	12.5 (0.0, 49.0)
Toppings, servings/d^a^	0.0 (0.0, 0.39)	0.09 (0.0, 0.67)	0.31 (0.0, 0.89)	0.0 (0.0, 0.80)	0.0 (0.0, 0.76)
SSBs, g/d^a^	0.0 (0.0, 100)	75.0 (0.0, 175)	75.0 (0.0, 200)	125 (0.0, 275)	200 (0.0, 375)
Juice, g/d^a^	0.0 (0.0, 100)	0.0 (0.0, 150)	0.0 (0.0, 100)	0.0 (0.0, 150)	0.0 (0.0, 150)
Fruits, g/d^a^	42.7 (5.7, 84.5)	57.1 (14.5, 103)	42.5 (12.5, 98.0)	31.7 (4.4, 100)	28.0 (2.0, 64.5)

*The categorical variables are expressed as percentages. The continuous variables are expressed as the means (SDs) unless stated otherwise*.

a *data is expressed as median (IQR) due to skewed distribution*.

*U-sugars, sum of urinary sucrose and fructose; LTPA, leisure-time physical activity; BMI, body mass index; U-osm, urine osmolality, SSB, sugar-sweetened beverage*.

### Correlations Between Urinary and Dietary Sugars

**Figure 2:**
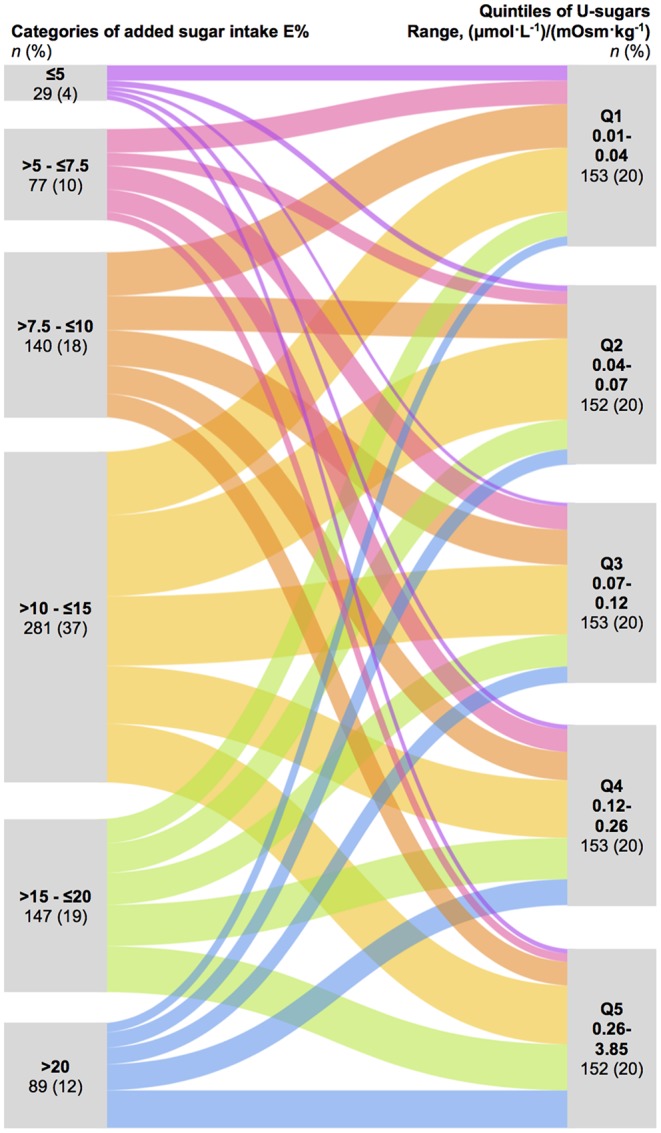
Alluvial plot demonstrating the agreement based on crosstabulation of the 6 categories of reported added sugar intake ( ≤ 5E, >5- ≤ 7.5E, >7.5- ≤ 10E, >10- ≤ 15E, >15- ≤ 20E, and >20E%) and quintiles of U-sugars (Q1–Q5). U-sugars, sum of urinary sucrose and fructose.

The alluvial plot in Figure 2 displays the agreement between the 6 categories of added sugar intake and quintiles of U-sugars based on the proportion of participants belonging to each category of the two different variables. In assessment of misclassification, the percentage of gross misclassification equaled 8% for women and 7% for men, while 32 and 34%, respectively, of the values were correctly classified (Supplementary Figure 1).

Sucrose intake (g/d), total sugar intake (g/d), total sugar density (g/1,000 kcal) and added sugar intake (E%) showed a higher statistically significant correlation with both U-sucrose and U-sugars (*r*≈0.20–0.30, *P* < 0.01), than with U-fructose (*r*≈0.11–0.14, *P* < 0.03), after adjusting for energy intake, age, sex and BMI (Table 3). Overall, the correlations were slightly weaker with total sugar and total sugar density (*r* = 0.21, *P* < 0.01 and *r* = 0.20, *P* < 0.01 with U-sugars, respectively) than with reported intake of sucrose and added sugar (both *r* = 0.24, *P* < 0.01 with U-sugars), and were weaker for women than for men e.g., (*r* = 0.20, *P* < 0.01 and *r* = 0.27, *P* < 0.01 between added sugar intake and U-sugars, respectively). U-sugars correlated with intake of desserts, sweets and SSBs, but not with toppings, juice and fruits. Additionally, among men but not women, SSB intake correlated with all the different urinary sugars, and U-fructose was positively correlated with juice intake and negatively correlated with fruit intake.

**Table 3:** Partial correlations between U-sucrose, U-fructose and U-sugars and different measures and sources of dietary sugars in all, women and men in the Malmö Offspring Study.

	**U-sucrose**			**U-fructose**			**U-sugars**		
	***n***	***r***	***P*-value**	***n***	***r***	***P*-value**	***n***	***r***	***P*-value**
**All**									
Sucrose (g/d)	889	0.27	<0.01	775	0.13	<0.01	763	0.24	<0.01
Total sugar (g/d)	889	0.22	<0.01	775	0.12	<0.01	763	0.21	<0.01
Total sugar density (g/1,000 kcal)	889	0.22	<0.01	775	0.13	<0.01	763	0.20	<0.01
Added sugar (E%)	889	0.27	<0.01	775	0.14	<0.01	763	0.24	<0.01
Desserts (g/d)	889	0.09	<0.01	775	0.05	0.14	763	0.11	<0.01
Sweets (g/d)	889	0.20	<0.01	775	0.07	0.04	763	0.18	<0.01
Toppings (servings/d)	889	0.03	0.31	775	−0.01	0.78	763	0.02	0.67
SSBs (g/d)	889	0.18	<0.01	775	0.09	0.01	763	0.16	<0.01
Juice (g/d)	889	0.04	0.25	775	0.11	<0.01	763	0.06	0.08
Fruits (g/d)	889	−0.04	0.21	775	−0.04	0.25	763	−0.05	0.15
**Women**									
Sucrose (g/d)	467	0.23	<0.01	421	0.11	0.03	412	0.19	<0.01
Total sugar (g/d)	467	0.19	<0.01	421	0.13	<0.01	412	0.18	<0.01
Total sugar density (g/1,000 kcal)	467	0.15	<0.01	421	0.12	0.02	412	0.16	<0.01
Added sugar (E%)	467	0.21	<0.01	421	0.13	<0.01	412	0.20	<0.01
Desserts (g/d)	467	0.05	0.25	421	0.005	0.92	412	0.09	0.07
Sweets (g/d)	467	0.21	<0.01	421	0.12	0.02	412	0.20	<0.01
Toppings (servings/d)	467	−0.02	0.65	421	−0.05	0.33	412	−0.04	0.48
SSBs (g/d)	467	0.08	0.08	421	0.01	0.79	412	0.05	0.34
Juice (g/d)	467	0.02	0.71	421	0.05	0.27	412	0.03	0.48
Fruits (g/d)	467	−0.05	0.33	421	0.0007	0.99	412	−0.03	0.60
**Men**									
Sucrose (g/d)	422	0.30	<0.01	354	0.14	<0.01	351	0.28	<0.01
Total sugar (g/d)	422	0.25	<0.01	354	0.11	0.03	351	0.22	<0.01
Total sugar density (g/1,000 kcal)	422	0.27	<0.01	354	0.12	0.02	351	0.23	<0.01
Added sugar (E%)	422	0.31	<0.01	354	0.13	0.01	351	0.27	<0.01
Desserts (g/d)	422	0.13	<0.01	354	0.11	0.05	351	0.12	0.02
Sweets (g/d)	422	0.18	<0.01	354	0.01	0.83	351	0.16	<0.01
Toppings (servings/d)	422	0.08	0.12	354	0.02	0.68	351	0.05	0.34
SSBs (g/d)	422	0.26	<0.01	354	0.16	<0.01	351	0.25	<0.01
Juice (g/d)	422	0.05	0.33	354	0.15	<0.01	351	0.09	0.11
Fruits (g/d)	422	−0.04	0.37	354	−0.12	0.03	351	−0.10	0.05

*The partial correlations are adjusted for age, sex, energy intake and BMI (not adjusted for sex in sex-specific analyses). The urinary sugar variables are log_10_-transformed*.

*U-sucrose, urinary sucrose; U-fructose, urinary fructose; U-sugars, sum of urinary sucrose and fructose; SSB, Sugar-sweetened beverages; BMI, body mass index*.

### Cardiometabolic Risk Factors

Differences between men and women were observed in the associations between sugar exposure (both added sugar intake and U-sugars) and several cardiometabolic risk factors. U-sugars, but not reported added sugar intake, were positively associated with systolic BP, diastolic BP and fasting glucose only in women. However, added sugar intake, but not U-sugars, was negatively associated with fasting glucose in men. Additionally, in women, both U-sugars and added sugar intake associated positively with BMI and waist circumference, whereas among men, U-sugars were negatively associated with BMI and waist circumference, and no association was observed for added sugar intake. Statistically significant interactions with sex were found for the associations of both U-sugars and added sugar intake with BMI and waist circumference. Added sugar intake was negatively associated with HDL cholesterol in both men and women. No associations were found with total cholesterol, triglycerides or LDL cholesterol (Table 4).

**Table 4:** Linear regression of U-sugars, added sugar intake and their composite measure (PC) on cardiometabolic risk factors in the Malmö Offspring Study.

	**All**				**Women**			**Men**		
	***n***	**ß**	**95% CI**	***P*-int sex**	***n***	**ß**	**95% CI**	***n***	**ß**	**95% CI**
**BMI (kg/m**^**2**^**)**										
U-sugars (μmol·L^−1^)/(mOsm·kg^−1^)										
Model 1	763	0.15	−0.45, 0.77		412	1.03	0.14, 1.92	351	−1.00	−1.84, −0.16
Model 2	677	0.08	−0.58, 0.74	<0.01	381	1.05	0.12, 1.97	296	−1.45	−2.40, −0.51
Added sugar (E%)										
Model 1	991	0.08	0.02, 0.13		533	0.14	0.06, 0.22	458	0.01	−0.05, 0.08
Model 2	889	0.03	−0.03, 0.09	0.03	493	0.10	0.01, 0.19	396	−0.03	−0.10, 0.05
Composite measure										
Model 1	763	0.35	0.15, 0.55		412	0.55	0.28, 0.82	351	−0.04	−0.35, 0.27
Model 2	677	0.26	0.04, 0.48	<0.01	381	0.50	0.22, 0.79	296	−0.24	−0.59, 0.11
**Waist circumference (cm)**										
U-sugars (μmol·L^−1^)/(mOsm·kg^−1^)										
Model 1	763	−0.09	−1.68, 1.50		412	2.00	−0.21, 4.21	351	−2.94	−5.18, −0.70
Model 2	677	−0.20	−1.84, 1.45	<0.01	381	2.02	−0.23, 4.28	296	−3.79	−6.19, −1.39
Added sugar (E%)										
Model 1	991	0.24	0.11, 0.37		533	0.34	0.14, 0.53	458	0.13	−0.05, 0.30
Model 2	889	0.15	0.01, 0.29	0.05	493	0.25	0.03, 0.46	396	0.05	−0.14, 0.24
Composite measure										
Model 1	763	1.00	0.49, 1.51		412	1.34	0.68, 2.00	351	0.23	−0.60, 1.06
Model 2	677	0.76	0.22, 1.30	<0.01	381	1.19	0.51, 1.88	296	0.19	−1.09, 0.70
**Total cholesterol (mmol/L)**										
U-sugars (μmol·L^−1^)/(mOsm·kg^−1^)										
Model 1	763	−0.08	−0.22, 0.05		412	−0.06	−0.23, 0.11	351	−0.11	−0.33, 0.11
Model 2	677	−0.14	−0.28, 0.01	0.91	381	−0.12	−0.30, 0.06	296	−0.15	−0.40, 0.10
Added sugar (E%)										
Model 1	990	−0.006	−0.02, 0.01		532	−0.009	−0.02, 0.01	458	−0.002	−0.02, 0.02
Model 2	888	−0.009	−0.02, 0.004	0.41	492	−0.02	−0.03, 0.0003	396	−0.001	−0.02, 0.02
Composite measure										
Model 1	763	−0.02	−0.06, 0.03		412	−0.02	−0.07, 0.04	351	−0.01	−0.09, 0.07
Model 2	677	−0.03	−0.07, 0.02	0.69	381	−0.03	−0.08, 0.03	296	−0.02	−0.11, 0.08
**Triglycerides (mmol/L)**										
U-sugars (μmol·L^−1^)/(mOsm·kg^−1^)										
Model 1	757	0.04	−0.05, 0.13		409	0.02	−0.07, 0.11	348	0.06	−0.11, 0.23
Model 2	671	0.02	−0.08, 0.11	0.96	378	0.007	−0.09, 0.11	293	−0.01	−0.20, 0.18
Added sugar (E%)										
Model 1	980	0.007	−0.001, 0.01		527	−0.00001	−0.01, 0.01	453	0.01	−0.001, 0.02
Model 2	878	0.003	−0.01, 0.01	0.49	487	−0.004	−0.01, 0.01	391	0.006	−0.01, 0.02
Composite measure										
Model 1	757	0.03	−0.00001, 0.06		409	0.01	−0.02, 0.04	348	0.06	−0.01, 0.12
Model 2	671	0.02	−0.01, 0.05	0.49	378	0.003	−0.03, 0.03	293	0.03	−0.03, 0.10
**HDL cholesterol (mmol/L)**										
U-sugars (μmol·L^−1^)/(mOsm·kg^−1^)										
Model 1	763	−0.06	−0.13, −0.003		412	−0.10	−0.19, −0.01	351	−0.02	−0.10, 0.06
Model 2	677	−0.04	−0.11, 0.02	0.16	381	−0.07	−0.17, 0.02	296	0.01	−0.07, 0.09
Added sugar (E%)										
Model 1	990	−0.02	−0.02, −0.01		532	−0.02	−0.03, −0.01	458	−0.01	−0.02, −0.005
Model 2	888	−0.01	−0.02, −0.01	0.04	492	−0.02	−0.02, −0.01	396	−0.009	−0.02, −0.002
Composite measure										
Model 1	763	−0.04	−0.06, −0.02		412	−0.05	−0.08, −0.02	351	−0.03	−0.06, −0.01
Model 2	677	−0.03	−0.05, −0.01	0.32	381	−0.03	−0.06, −0.001	296	−0.03	−0.06, 0.002
**LDL cholesterol (mmol/L)**										
U-sugars (μmol·L^−1^)/(mOsm·kg^−1^)										
Model 1	763	−0.06	−0.18, 0.06		412	−0.02	−0.17, 0.13	351	−0.11	−0.31, 0.09
Model 2	677	−0.13	−0.26, 0.01	0.66	381	−0.09	−0.26, 0.07	296	−0.16	−0.39, 0.07
Added sugar (E%)										
Model 1	989	0.006	−0.004, 0.02		531	0.006	−0.01, 0.02	458	0.007	−0.01, 0.02
Model 2	887	0.002	−0.01, 0.01	0.68	491	−0.003	−0.02, 0.01	396	0.008	−0.01, 0.03
Composite measure										
Model 1	763	0.01	−0.03, 0.05		412	0.02	−0.03, 0.06	351	0.006	−0.07, 0.08
Model 2	677	−0.006	−0.05, 0.04	0.90	381	−0.006	−0.06, 0.04	296	0.008	−0.07, 0.09
**Systolic BP (mmHg)**										
U-sugars (μmol·L^−1^)/(mOsm·kg^−1^)										
Model 1	761	2.92	1.13, −4.70		410	4.22	1.74, 6.71	351	1.54	−0.98, 4.05
Model 2	675	2.95	0.99, 4.92	0.22	379	4.63	1.96, 7.30	296	1.30	−1.55, 4.16
Added sugar (E%)										
Model 1	988	−0.09	−0.24, 0.05		530	−0.02	−0.23, 0.20	458	−0.12	−0.31, 0.07
Model 2	886	−0.09	−0.26, 0.08	0.87	490	−0.02	−0.27, 0.22	396	−0.09	−0.31, 0.13
Composite measure										
Model 1	761	0.48	−0.11, 1.06		410	0.89	0.13, 1.66	351	0.13	−0.79, 1.06
Model 2	675	0.46	−0.19, 1.12	0.55	379	1.01	0.17, 1.85	296	−0.09	−1.13, 0.96
**Diastolic BP (mmHg)**										
U-sugars (μmol·L^−1^)/(mOsm·kg^−1^)										
Model 1	761	1.10	−0.13, 2.33		410	1.80	0.16, 3.43	351	0.17	−1.71, 2.04
Model 2	675	1.30	−0.01, 2.62	0.43	379	1.81	0.07, 3.55	296	0.58	−1.48, 2.64
Added sugar (E%)										
Model 1	988	0.03	−0.07, 0.13		530	0.008	−0.13, 0.15	458	0.05	−0.09, 0.20
Model 2	886	−0.01	−0.12, 0.10	0.98	490	−0.02	−0.18, 0.14	396	0.003	−0.15, 0.16
Composite measure										
Model 1	761	0.54	0.14, 0.94		410	0.49	−0.01, 0.99	351	0.60	−0.08, 1.28
Model 2	675	0.45	0.02, 0.88	0.86	379	0.48	−0.06, 1.02	296	0.41	−0.34, 1.16
**Fasting glucose (mmol/L)**										
U-sugars (μmol·L^−1^)/(mOsm·kg^−1^)										
Model 1	762	0.09	−0.003, 0.19		412	0.14	0.04, 0.25	350	0.01	−0.15, 0.18
Model 2	677	0.10	0.0003, 0.20	0.22	381	0.16	0.05, 0.26	296	0.03	−0.17, 0.22
Added sugar (E%)										
Model 1	990	−0.007	−0.01, 0.001		533	−0.003	−0.01, 0.01	457	−0.01	−0.02, −0.00001
Model 2	889	−0.008	−0.02, 0.001	0.04	493	−0.002	−0.01, 0.01	396	−0.01	−0.03, 0.001
Composite measure										
Model 1	762	−0.0003	−0.03, 0.03		412	0.009	−0.02, 0.04	350	−0.03	−0.09, 0.03
Model 2	677	−0.0004	−0.03, 0.03	0.11	381	0.01	−0.02, 0.05	296	−0.03	−0.10, 0.04

*Total cholesterol, triglycerides, HDL, and LDL cholesterol and fasting glucose are measured in plasma. U-sugars are log_10_-transformed. The composite measure is the first PC of the two variables U-sugars and added sugars*.

*Model 1 is adjusted for age and sex (and energy intake for added sugar and the composite measure)*.

*Model 2 is additionally adjusted for educational level, LTPA, smoking status, alcohol habits, and fiber density. Regressions with total cholesterol, triglycerides, HDL, and LDL cholesterol are additionally adjusted for usage of lipid lowering drugs and regressions with systolic and diastolic BP are additionally adjusted for usage of antihypertensive drugs*.

*U-sugars, sum of urinary sucrose and fructose; PC, principal component; BMI, body mass index; HDL, high-density lipoprotein; LDL, low-density lipoprotein; BP, blood pressure; LTPA, leisure-time physical activity*.

The combination of U-sugars and reported added sugars into a composite measure of sugar exposure using the PC method revealed statistically significant positive associations with BMI, waist circumference and systolic BP, and a statistically significant negative association with HDL cholesterol in women, whereas none of the cardiometabolic risk factors were associated with the PC of sugar exposure in men (Table 4). Combining by Howe's method (Supplementary Table 1) showed resembling associations with the same cardiometabolic risk factors as when using the PC method, but overall yielded lower coefficients and only the associations with HDL were statistically significant.

Energy underreporting was identified as a statistically significant effect modifier in the associations between reported added sugar intake and BMI and waist circumference in women. These positive associations were attenuated after the removal of energy underreporters. A statistically significant interaction between obesity and U-sugars was obtained in the regression with systolic BP in women: obese individuals exhibited a markedly stronger positive association than the non-obese individuals (BMI≥30: β = 3.15, *P* = 0.03; BMI <30: β = 11.9, *P* < 0.01; Supplementary Table 2).

### Potential Predictors of Overnight Urinary Sugars

We used stepwise backward linear regression in an attempt to identify the major predictors of U-sugars (not adjusted for U-osm) (Table 5). After taking possible and measured predictors into account, the main predictors of the various sugar intake variables were added sugar intake for men (*r* = 0.31) and intake of desserts (*r* = 0.10) and sweets (*r* = 0.21) for women. U-osm was found to be a strong predictor in both women and men (*r* = 0.41 and *r* = 0.40, respectively). Systolic BP and fasting glucose also exhibited positive associations with U-sugars in women, whereas education level and waist circumference showed negative associations with U-sugars in men. When examining osmolality-adjusted U-sugars instead, excluding U-osm in the multivariate model, the same predictors remained to a similar extent (data not shown).

**Table 5:** Partial correlation coefficients between U-sugars (not adjusted for U-osm) and its potential predictors in women and men of the Malmö Offspring Study.

	**Women (*****n*** **=** **373)**				**Men (*****n*** **=** **295)**			
	**Separate models**^****a****^		**Multivariate model**^**a**^^**,**^ ^**b**^		**Separate models**^****a****^		**Multivariate model**^**a**^^**,**^ ^**b**^	
	***r***	***P***	***r***	***P***	***r***	***P***	***r***	***P***
Added sugar (E%)	0.23	<0.01			0.27	<0.01	0.31	<0.01
Desserts (g/d)	0.09	0.08	0.10	0.04	0.14	<0.01		
Sweets (g/d)	0.22	<0.01	0.21	<0.01	0.16	<0.01		
Toppings (servings/d)	−0.02	0.72			0.05	0.35		
SSBs (g/d)	0.07	0.15			0.25	<0.01		
Fruits (g/d)	−0.10	0.05			−0.14	0.01		
Juice (g/d)	0.02	0.68			0.10	0.07		
Education level	−0.12	0.03			−0.12	0.05	−0.13	0.02
Smoking status	0.05	0.35			0.06	0.28		
Alcohol habits	−0.09	0.09			−0.005	0.93		
LTPA	−0.05	0.34			−0.03	0.56		
BMI (kg/m^2^)	0.11	0.02			−0.08	0.12		
Waist circumference (cm)	0.09	0.08			−0.08	0.12	−0.18	<0.01
Systolic BP (mmHg)	0.18	<0.01	0.17	<0.01	0.08	0.13		
Fasting glucose (mmol/L)	0.13	<0.01	0.12	0.01	0.01	0.78		
U-osm (mOsm/kg)	0.41	<0.01	0.41	<0.01	0.39	<0.01	0.40	<0.01
e-GFR (ml/min/1.73 m^2^)	−0.05	0.33			−0.08	0.16		

a *All partial correlations are adjusted for age and energy intake*.

b *The multivariate partial correlation model was determined through stepwise backward linear regression. All covariates were added simultaneously to a linear regression model and the covariate with the highest P-value was excluded in a stepwise manner from the model until all covariates were deemed significant*.

*U-sugars are log_10_-transformed. Fasting glucose is measured in plasma*.

*U-sugars, sum of urinary sucrose and fructose; U-osm, urine osmolality; SSB, sugar-sweetened beverages; LTPA, leisure-time physical activity; BMI, body mass index; BP, blood pressure; e-GFR, estimated glomerular filtration rate*.

## Discussion

We showed statistically significant correlations of *r*≈0.20–0.30 between reported sugar intakes and overnight urinary sugars after adjusting for age, sex, energy intake, and BMI. The relatively low coefficients of these correlations and the modest agreement observed in the alluvial plot may reflect that both these measures of sugar intake are subject to random variation and measurement error. However, importantly, such possible errors are completely unrelated (misreporting and potential unknown determinants), which therefore indicates a potential and need for the combination of these two measurements. As discussed and shown in both data simulations and in real world examples by Freedman et al. (17, 18), even when the correlation between reported intakes and the biomarker is not very high, combination of the two measurements is motivated. Hence, these two measurements could potentially complement each other to improve the assessment of the associations between added sugar intake and cardiometabolic risk.

The observed correlation coefficients in this study agree with the results of a previous study of spot morning urine samples in children (*r* = 0.25) (13). This similarity was obtained even though the collection of urinary and dietary data did not reflect the exact same days in our study, which was the case in the previous study. However, the previous study did not reveal large differences in the comparison of the single 24-h recall from the day before collection of the morning spot urine samples and multiple 24-h recalls (13). The correlation coefficients obtained in our study are also similar to previous findings obtained with the validated predictive 24-h urinary sugar biomarker in free living populations; in the Nutrition and Physical Activity Assessment Study, the correlation with total sugar density from a 4-day food record was *r* = 0.21 (8), which is comparable to that found in our study between total sugar density and U-sugars (*r* = 0.20). Because we only can compare against self-reported sugar intake and not true intake, it is not straightforward to compare these correlations and the observed exact agreement of 32–34% to warranted limits used in biomarker validation studies [*r* = 0.5–0.6 and quartile agreement of at least 50% (28, 29)].

In our study, the correlation of reported added sugar intake with U-fructose was notably weaker than that with U-sucrose (0.14 and 0.27, respectively), even though only monosaccharides, in theory, should be absorbed in the jejunum. In addition, total sugar (g/d) and total sugar density (g/1,000 kcal) were not as strongly correlated with the urinary sugars as sucrose intake (g/d) and added sugar intake (E%). Previous studies have also revealed weaker correlations for intrinsic sugar (included in total sugars) than for extrinsic sugars (mainly added sugars) with the urinary sugar biomarker (13, 30). This effect could be due to the rate of digestion and absorption of the sugars, which is believed to be lower when the sugars are naturally occurring in complex foods compared to simple sugars added to foods (31). Additionally, the relatively high intakes of sugars from dairy (lactose, included in total sugars) in the Swedish diet might contribute to some of these differences.

We observed slightly stronger correlations between dietary sugars and urinary sugars in men than in women, which is supported by the findings of previous studies (7, 12). In addition to biological differences between men and women, another plausible reason for the sex differences could be that women generally tend to underreport their dietary intake more than men (7, 32, 33). However, we do not know the degree of sugar intake underreporting in our study, and energy misreporting does not necessarily reflect misreporting of sugar. However, the percentage of energy underreporters was lower among those with higher added sugar intake and tended to be lower in those with higher U-sugars. Furthermore, the high proportion of zero-reporters of SSB intake among women (57%), might contribute to why we only can see a statistically significant correlation between SSB intake and U-sugars in men but not in women.

No previous studies have evaluated urinary sugar biomarkers in relation to cardiometabolic risk factors other than anthropometric measurements. The examination of spot urinary sucrose (not morning urine) in relation to obesity measures in the EPIC Norfolk cohort revealed a positive association between the risk of being overweight and higher spot urinary sucrose, whereas a negative association between risk of being overweight and higher self-reported sugar intake (12). Hence, it can be speculated that the lack of a positive association between reported sugar intake and risk of being overweight might be partly explained by a measurement error bias in the dietary assessment, which is not an issue with the objective measurement of sucrose in spot urine samples. Similar patterns were observed in our study for systolic BP, diastolic BP and fasting glucose in women; these parameters were positively associated with U-sugars but not with reported added sugar intake. However, such a pattern was not observed for the other cardiometabolic risk factors. In fact, both U-sugars and added sugar intake were positively associated with BMI and waist circumference and negatively associated with HDL cholesterol in women, indicating quite credible associations, and the combination of the two measurements strengthened the associations for these cardiometabolic risk factors. In men, however, U-sugars were negatively associated with BMI and waist circumference, while no association was found with added sugar intake. Nevertheless, cross-sectional examination of BMI and waist circumference in relation to dietary intake is difficult because large body measurements might affect one's dietary awareness more than the “nonvisual” cardiometabolic risk factors. Hence, the direction of the observed associations is uncertain. Furthermore, there may exist sex differences in the effects on weight gain from a high sugar diet as it has been observed that the inhibition of lipolysis by insulin is more profound in women than men (34). Our observed sex differences in the associations with cardiometabolic risk factors were unexpected findings outside the scope of our study objective, which futures studies are encouraged to elucidate.

Previous studies have discussed whether the amount of urinary sucrose and fructose might differ between obese and lean participants (11, 35) due to the potentially higher gut permeability of obese individuals (36, 37). Therefore, the associations between U-sugars and measures of obesity might be due to other underlying causes in addition to the notion that a high sugar intake would lead to weight gain. Nevertheless, no difference in the 24-h urinary levels of either sucrose or fructose in obese compared with normal weight subjects was observed in a randomized controlled trial (35). In women, we observed positive correlations between U-sugar and systolic BP and fasting glucose and these parameters also fell out as predictors of U-sugars, but one could discuss the putative causal direction of these associations. In addition to the theory that a high sugar intake would lead to an impaired metabolic status, both systolic BP and fasting glucose are major risk factors for renal insufficiency and it is possible that this could influence urinary excretion of sugars (38). However, to our knowledge, no previous study has shown that the amounts of sucrose and fructose excreted in the urine is affected by insulin resistance. Because only glucose is regulated by insulin in the circulation, the same principle as for urinary excretion of glucose cannot be applied to sucrose and fructose.

The limitations of this study are the lack of longitudinal data for the cardiometabolic risk factors and that urinary sugar data is generated from overnight urine samples instead of 24-h samples. We were therefore also bound to use other methods than regression calibration for combining the biomarker with reported intake (10). To date, the overnight urine biomarker has only been compared to self-reported sugar intake data, which cannot be used for validating a nutritional biomarker (39), and no earlier study has ever reported the correlation between the sugar concentrations in overnight urine samples and 24-h urine samples. However, the benefit of using overnight samples over any time spot samples is that they are less affected by recent past meals (40). Furthermore, residual confounding can almost be considered indisputable, and future studies are needed to identify the determinants of spot and overnight urinary sugars. Therefore, the following important question remains: Which measurement is most valid, the self-reported added sugar intake, which is likely to be biased by misreporting, or the sum of sucrose and fructose in overnight urine samples, which only reflects a point measurement and for which determinants other than sugar intake remain unknown? At this current state of knowledge, we believe that they both contribute partly to the truth and may complement each other. However, this must be validated against true sugar intake or the 24-h urinary sucrose and fructose biomarker in the future. Future studies should also investigate potential sex differences to improve the understanding of the urinary sugar biomarker, as well as considering the use of repeated overnight or spot urine samples to obtain improved precision, while still facilitating urine collection as compared with 24-h urine sampling.

In summary, we found statistically significant correlations at levels of *r*≈0.20–0.30 and demonstrated the potential for using the sugar level in overnight urine samples to complement self-reported dietary data in investigations of cardiometabolic risk. The combination of U-sugars and added sugar intake indicated that a higher sugar intake in women is associated with higher BMI, waist circumference and systolic BP and lower HDL cholesterol. Considering the potential gains from collecting only overnight urine instead of 24-h urine in regard to participant burden, drop-out rates, missing data and selective participation, the overnight urinary sugar biomarker calls for further validation.

## Data Availability Statement

The dataset for this article is not publicly available because of ethical and legal restrictions. Requests to access the dataset should be directed to the Chair of the Steering Committee for the Malmö cohorts, see instructions at https://www.malmo-kohorter.lu.se/english.

## Ethics Statement

The studies involving human participants were reviewed and approved by the Regional Ethics Committee in Lund. The patients/participants provided their written informed consent to participate in this study.

## Author Contributions

SR and ES designed the research. SR performed all statistical analyses and wrote the manuscript under the supervision of ES. NG performed the laboratory analyses of the urine samples under the supervision of GK. SE contributed with data on the urine osmolality. The collection of dietary data in the MOS was administered by SH, UE, LB, and SR. PN is the principal investigator for the MOS, whereas MO-M is in charge of the dietary data in the MOS. NG, SH, LB, SE, GE, PN, MO-M, UE, GK, and ES all contributed important input to the manuscript. SR and ES had primary responsibility for the final content.

## Conflict of Interest

GK previously received an unrestricted grant from Mars for research on flavanols unrelated to the current study. The remaining authors declare that the research was conducted in the absence of any commercial or financial relationships that could be construed as a potential conflict of interest.

## Abbreviations

BMI: body mass index
BP: blood pressure
e-GFR: estimated glomerular filtration rate
HDL: high-density lipoprotein
LDL: low-density lipoprotein
LTPA: leisure-time physical activity
MDC-CC: malmö diet and cancer-cardiovascular cohort
MOS: malmö offspring study
PC: principal component
QC: quality control
SSB: sugar-sweetened beverage
U-fructose: urinary fructose
U-osm: urine osmolality
U-sucrose: urinary sucrose
U-sugars: sum of urinary sucrose and fructose.
