# Supplementary material for: Comparing Self-Reported Sugar Intake With the Sucrose and Fructose Biomarker From Overnight Urine Samples in Relation to Cardiometabolic Risk Factors
Source: Front Nutr. 2020 May 6;7:62. doi: 10.3389/fnut.2020.00062 (PMC7218081; doi:10.3389/fnut.2020.00062)
Supplement: Supplementary file 1 [file Data_Sheet_1.pdf]

frontiers

Supplementary Material

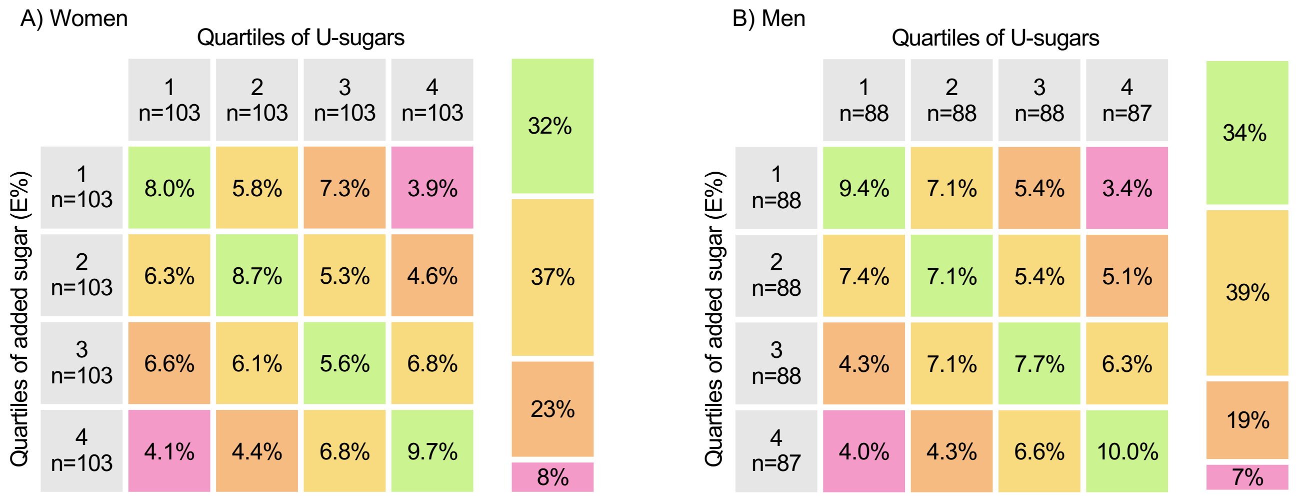

Supplementary Figure 1. Assessment of misclassification by crosstabulation of quartiles of U-sugars and added sugar intake (n=763) for A) women and B) men. Green represent those classified in the correct quartile. Yellow represent those classified adjacent to the correct quartile. Orange represent those classified adjacent to the opposite quartile. Pink represent those classified in opposite quartiles. The bar on the right shows the total distribution belonging to each category. U-sugars=Sum of Urinary Sucrose and Fructose.

Supplementary Table 1. Linear regression of combined measures using Howe's method of U-sugars and added sugar intake on cardiometabolic risk factors

	All			Women			Men		
	n	ß	*P*	*n*	ß	*P*	*n*	ß	*P*
BMI (kg/m^2)									
Model 1	763	0.0005	0.257	412	0.002	0.026	351	-0.0007	0.262
Model 2	677	-0.00002	0.964	381	0.001	0.114	296	-0.0014	0.036
Waist circumference (cm)									
Model 1	763	0.002	0.143	412	0.004	0.023	351	-0.001	0.534
Model 2	677	0.0005	0.713	381	0.003	0.116	296	-0.003	0.133
Total cholesterol (mmol/L)									
Model 1	763	-0.0001	0.270	412	-0.0001	0.353	351	-0.0001	0.572
Model 2	677	-0.0002	0.093	381	-0.0002	0.110	296	-0.0001	0.523
Triglycerides (mmol/L)									
Model 1	757	0.0001	0.056	409	0.00005	0.495	348	0.0002	0.110
Model 2	671	0.0001	0.325	378	$5.7e^{-6}$	0.945	293	0.00008	0.525
HDL cholesterol (mmol/L)									
Model 1	763	-0.0002	<0.001	412	-0.0003	<0.001	351	-0.0001	0.041
Model 2	677	-0.0001	0.005	381	-0.0002	0.013	297	-0.00007	0.206
LDL cholesterol (mmol/L)									
Model 1	763	$1.5\ e^{-6}$	0.987	412	0.00004	0.734	351	-0.00003	0.828
Model 2	671	-0.0001	0.317	381	-0.00001	0.360	296	-0.00005	0.749
Systolic BP (mmHg)									
Model 1	761	0.002	0.194	410	0.003	0.083	351	0.0009	0.628
Model 2	675	0.002	0.270	379	0.004	0.055	296	0.00006	0.975
Diastolic BP (mmHg)									
Model 1	761	0.002	0.085	410	0.001	0.268	351	0.002	0.218
Model 2	675	0.001	0.270	379	0.001	0.420	296	0.0009	0.532
Fasting glucose (mmol/L)									
Model 1	762	-0.00002	0.741	412	0.00004	0.592	350	-0.0001	0.279
Model 2	678	-0.00001	0.864	381	0.00008	0.406	295	-0.0001	0.389

Model 1 is adjusted for age, sex and energy intake.
Model 2 is additionally adjusted for education, LTPA, smoking status, alcohol habits and fiber density.
BMI=Body Mass Index; BP=Blood Pressure; HDL=High-density Lipoprotein; LDL=Low-density Lipoprotein; LTPA=Leisure-time Physical Activity; U-sugars=Sum of Urinary Sucrose and Fructose

Supplementary Table 2. Evaluation of interactions and sensitivity analysis in linear regression analyses

	Men	Women				
	P-int	*P*-int	Sensitivity analysis	*n*	ß	*P*
BMI (kg/m^2)						
Added sugar × underreporting	0.895	0.013	No underreporters	*350*	*0.058*	*0.238*
Waist circumference						
Added sugar × underreporting	0.727	0.001	No underreporters	*350*	*0.084*	*0.465*
Total cholesterol (mmol/L)						
U-sugars × obesity	0.678	0.996				
Added sugar × underreporting	0.101	0.541				
Added sugar × obesity	0.118	0.759				
Triglycerides (mmol/L)						
U-sugars × obesity	0.837	0.080				
Added sugar × underreporting	0.187	0.988				
Added sugar × obesity	0.865	0.911				
HDL cholesterol (mmol/L)						
U-sugars × obesity	0.834	0.947				
Added sugar × underreporting	0.516	0.078				
Added sugar × obesity	0.801	0.598				
LDL cholesterol (mmol/L)						
U-sugars × obesity	0.592	0.653				
Added sugar × underreporting	0.073	0.699				
Added sugar × obesity	0.102	0.992				
Systolic BP (mmHg)						
U-sugars × obesity	0.834	0.049	No obese	*329*	*3.146*	*0.033*
Added sugar × underreporting	0.762	0.883				
Added sugar × obesity	0.301	0.839				
Diastolic BP (mmHg)						
U-sugars × obesity	0.916	0.599				
Added sugar × underreporting	0.389	0.933				
Added sugar × obesity	0.354	0.731				
Fasting glucose (mmol/L)						
U-sugars × obesity	0.900	0.435				
Added sugar × underreporting	0.081	0.797				
Added sugar × obesity	0.938	0.050	No obese	*424*	*-0.005*	*0.327*

In sex specific analysis, interaction with obesity (BMI≥30) were evaluated with U-sugars and interaction with underreporting of energy and obesity were evaluated with added sugar intake. However, obesity was not evaluated with BMI and WC. In case the interaction was significant, a stratified analysis was performed.
U-sugars are log_{10}-transformed.
Adjustment according to model 2 was conducted; age, (energy intake for added sugar), education, LTPA, smoking status, alcohol habits and fiber density.
BMI=Body Mass Index; BP=Blood Pressure; HDL=High-density Lipoprotein; LDL=Low-density Lipoprotein; LTPA=Leisure-time Physical Activity; U-sugars=Sum of Urinary Sucrose and Fructose.
